# Achilles tendon resting angle is able to detect deficits after an Achilles tendon rupture, but it is not a surrogate for direct measurements of tendon elongation, function or symptoms

**DOI:** 10.1007/s00167-022-07142-9

**Published:** 2022-09-10

**Authors:** Elin Larsson, Katarina Nilsson Helander, Lotta Falkheden Henning, Mervi Heiskanen, Michael R. Carmont, Karin Grävare Silbernagel, Annelie Brorsson

**Affiliations:** 1 The Department of Orthopaedics, Sahlgrenska University Hospital Mölndal, Institute of Clinical Sciences at Sahlgrenska Academy, Gothenburg University, Göteborgsvägen 31, 431 80 Mölndal, Sweden; 2 Department of Orthopaedics, Institute of Clinical Sciences at Sahlgrenska Academy, Gothenburg University, Gothenburg, Sweden; 3 Aktiva Rehab Team, Gothenburg, Sweden; 4 The Department of Trauma and Orthopaedic Surgery, Princess Royal Hospital, Shrewsbury and Telford Hospital NHS Trust, Shropshire, UK; 5 Department of Physical Therapy, University of Delaware, Newark, DE USA; 6 IFK Kliniken Rehab, Gothenburg, Sweden

**Keywords:** Achilles tendon rupture, Achilles tendon resting angle, ATRA, Tendon elongation, Tendon length, Ultrasound, Functional tests

## Abstract

**Purpose:**

The aim of this study was to investigate how the Achilles tendon resting angle (ATRA), an indirect measurement of tendon elongation, correlates with ultrasonography (US) measurements of the Achilles tendon length 6 and 12 months after an acute ATR and relates to other clinical outcome measurements such as heel-rise height, jumping ability and patient-reported outcome measurements (PROMs).

**Methods:**

Patients were included following acute Achilles tendon rupture (ATR). Achilles tendon length, ATRA, heel-rise height (HRH), drop countermovement jump (Drop CMJ) and PROMs (Achilles tendon total rupture score (ATRS) and physical activity scale (PAS)) were evaluated 6 and 12 months after injury. Achilles tendon length was evaluated using US, while the ATRA was measured with a goniometer.

**Results:**

Sixty patients (13 women, 47 men), mean (SD) age 43 (9) years, with an acute ATR undergoing either surgical (35%) or non-surgical (65%) treatment were evaluated. A negative correlation (*r* = − 0.356, *p* = 0.010) between relative ATRA and tendon elongation was seen at 12 months after ATR. There were also significant positive correlations at 6 and 12 months between relative ATRA and HRH (*r* = 0.330, *p* = 0.011 and *r* = 0.379, *p* = 0.004). There were no correlations between ATRA and ATRS or ATRA and Drop CMJ, at either 6 or 12 months after the injury.

**Conclusion:**

In combination with other clinical evaluations such as HRH and US, ATRA could be a clinical tool for indirect measurements of tendon elongation. However, ATRA cannot be recommended as a direct surrogate for US for determining Achilles tendon length.

**Level of evidence:**

III.

## Introduction

The question of how best to treat an acute Achilles tendon rupture (ATR), surgically or non-surgically, has been addressed in numerous studies, but there is still no consensus [13, 16, 17, 19]. The aim of treatment is to optimise the functional outcome and minimise the complications of the injury and treatment. Meta-analyses have concluded that the re-rupture rate is lower when ATR is treated surgically compared with non-surgical treatment [9, 20]. The risk of other complications, such as wound infection and iatrogenic nerve injury, is reported to be higher with surgical treatment, while the incidence of deep vein thrombosis (DVT) is similar in both treatment regimens [9, 20].

Tendon elongation is a complication after an ATR, leading to reduced plantar flexion strength and poor outcome [10]. Regardless of surgical or non-surgical treatment, the injured tendon elongates during the healing process [10]. Reduced tendon elongation correlates with superior clinical outcome [12]. There are several methods for assessing tendon length and elongation. Radiography with intra-tendinous markers and magnetic resonance imaging (MRI) have been used, but these methods are expensive and may not be accessible for monitoring during recovery [12, 15]. The ultrasonographic (US) measurement of tendon length has been validated and found to be a reliable and useful tool, but requires expertise and suitable ultrasound machines may not be readily available in clinics [26].

The Achilles tendon resting angle (ATRA) is a simple, readily available, less expensive means of determining the resting position of the ankle following ATR, with a low measurement error [27]. The ATRA is defined as “the angle between the long axis of the fibula and the line from the tip of the fibula to the head of the fifth metatarsal” [4]. The ATRA has been validated for assessing tendon elongation against US [28]. According to Carmont et al. [3], the absolute ATRA increases following injury, reduces as a result of surgery and increases again during the first phase of rehabilitation by 3 months. In previous studies, the ATRA has been reported to correlate with patient-reported outcome measurements (PROMs) at three and 6 months after surgical treatment [3]. In overall terms, the ATRA is a frequently used evaluation of tendon length in studies focusing on rehabilitation after ATR, as it has been considered to be more accessible at an earlier stage in the clinical practice than US [3–5, 7, 8, 28].

Like the ATRA, the heel-rise test is an easily performed evaluation of ankle plantar flexion function during rehabilitation. Studies evaluating recovery at 6 and 12 months often include measurements of both heel-rise endurance (repetitions or total work performed) and maximum heel-rise height [1–3, 21, 25]. The deficit in maximum heel-rise height (HRH), as compared with the uninjured side, has been found to correlate to the degree of tendon elongation.

The evaluation of tendon elongation and associated outcomes needs to be further explored to improve our clinical tools in order to optimise the rehabilitation and outcome after an ATR. It is important to find a clinical evaluation method that is valid and responsive to changes over time and correlates with tendon elongation (14). The aim of this study is therefore to determine how the ATRA correlates with the length of the Achilles tendon measured with US, HRH, drop countermovement jump (Drop CMJ) and the Achilles tendon total rupture score (ATRS) 6 and 12 months after an acute ATR.

## Materials and methods

The research protocol was approved by the Swedish Ethical Review Authority (Dnr 803–15).

All the subjects gave their written consent for enrolment after having been provided with oral and written information about the study.

The patients were recruited within 6 months following an acute ATR between 2016 and 2020 by physiotherapists at five different clinics in Gothenburg, Sweden. The inclusion criteria for the study were age 18–65 years and treatment commenced within 4 days of injury. The exclusion criteria included previous injury to the Achilles tendon on either leg, neurological disease or lack of comprehension of written and oral Swedish.

The patients were evaluated at 6 and 12 months following rupture and the same experienced physiotherapist performed all the evaluations.

### Achilles tendon resting angle (ATRA)

**Fig. 1:**
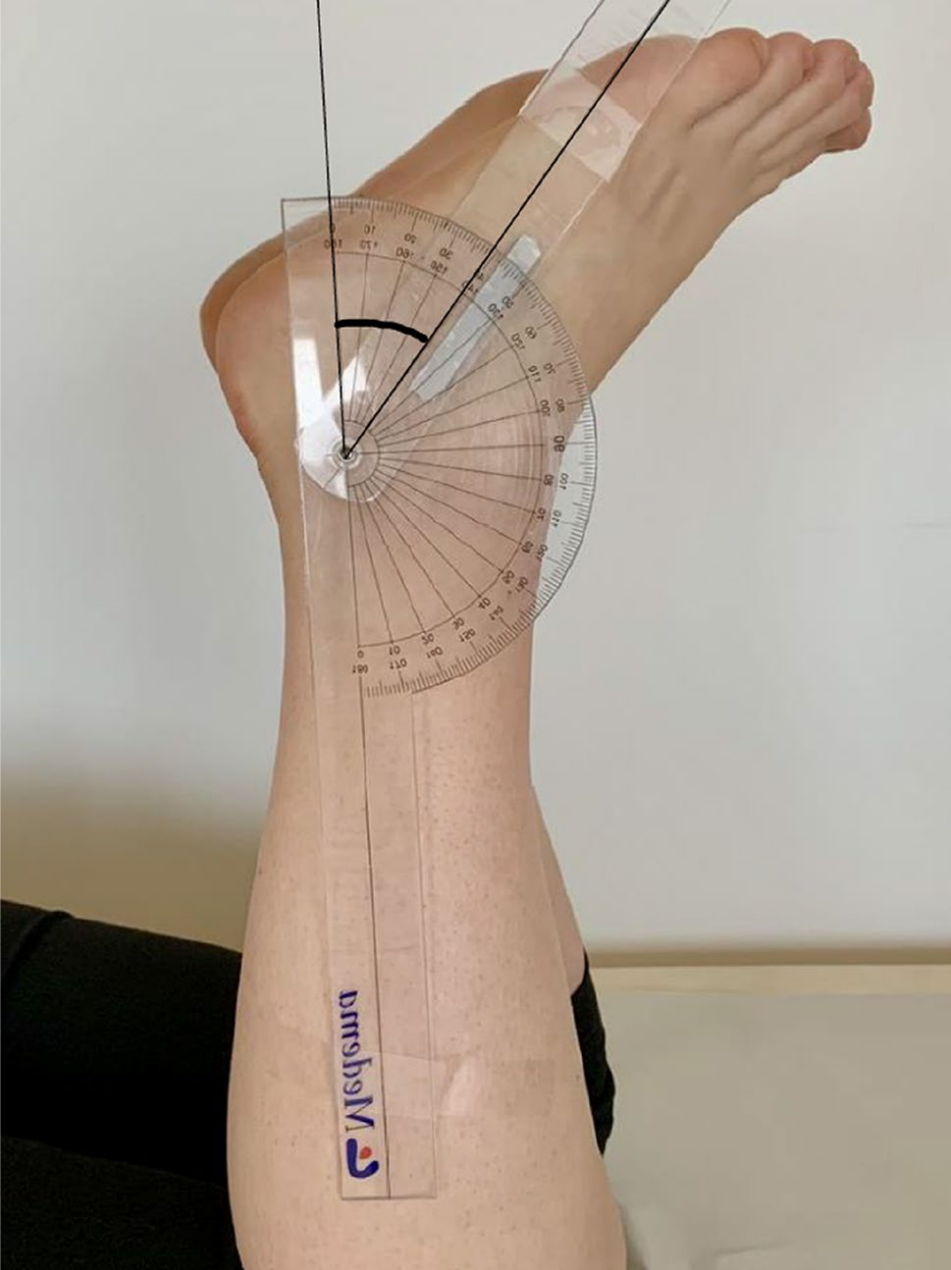
The Achilles tendon resting angle (ATRA) measured with a goniometer

For measurements of the ATRA, patients were positioned prone with their knee flexed to 90 degrees and encouraged to relax their ankle joints. The non-injured leg was examined first. The patients were encouraged to relax their ankle joints. The axis of the goniometer (1˚increments) was positioned at the tip of the fibula. One arm of the goniometer was aimed towards the head of the fibula and the other arm of the goniometer was positioned to bisect the head of the fifth metatarsal (Fig. 1). The ATRA has been reliability tested by Carmont et al. [4] and the reported ICC value of the ATRA is 0.91–0.92 [4].

The ATRA was reported as either the *absolute ATRA* of the injured foot or the *relative ATRA* referring to differentiation between the resting angle of the injured foot and the non-injured foot respectively. Increased dorsiflexion is considered to be a negative relative ATRA value and increased plantar flexion a positive ATRA value compared with the non-injured side [3].

### Ultrasonographic (US) measurement

Achilles tendon length was measured using a US extended field-of-view feature. The patients lay prone on the examination table with both feet hanging over the edge of the examination table. This enabled both feet to be equally relaxed during measurement. The length of the tendon was measured between the calcaneal notch, the most proximal attachment of the Achilles tendon on the calcaneum, and the gastrocnemius musculotendinous junction, with extended field-of-view US (Logiq E or Logiq P9 Ultrasound; GE Healthcare X AB), using a wideband array linear probe (5.0–13.0 MHz). The B-mode at 10 MHz and a depth of 3 cm were used to record the images. This has been found to be a valid and reliable method with an ICC value of 0.90 [26]. All the images were measured by a second experienced physiotherapist. The mean value of two to three images for each foot and patient was used for calculations. The tendon elongation was calculated as the value on the injured limb minus the value on the healthy limb and expressed in centimetres.

### Achilles tendon total rupture score (ATRS)

The evaluation of patient-reported symptoms and function was assessed using the Achilles tendon total rupture score (ATRS). The ATRS is a ten-item injury-specific and self-administered score with a maximum score of 100. The maximum score implies no limitations and a full recovery. The ATRS has been found to be a valid and reliable instrument for measuring the outcome related to symptoms and physical activity after ATR [18].

### Functional evaluations

Functional evaluations were made with a test battery that included the standing heel-rise work test to evaluate muscular endurance (Joule) and the height of the heel rise (cm) [23]. The single-leg heel rise, which is used in this study, is a reliable and valid clinical test for evaluating patients with ATR [24]. The Muscle Lab^®^ measurement system (Ergotest Technology, Oslo, Norway) was used for functional evaluations.

Before the evaluation, patients performed a 5-min warm-up on a stationary bike and performed three sets of ten two-legged heel rises. Standardised shoes (Bagheera Omega) were worn and the non-injured side was evaluated first.

In the single-leg heel-rise test, the patient stood on one foot on a box with a 10° incline and a linear encoder was attached to the shoe on the tested leg. The patients were instructed to lift as high as possible during the heel rise and keep their knee straight. The test continued for as long as patients were able to perform a heel rise of at least 2 cm height and maintain the tempo indicated by a metronome at a tempo of 30 heel rises a minute. For heel-rise height, the maximum height was used for calculations. Deficits in heel-rise height were defined as the difference between the injured and the non-injured side expressed in cm.

A further functional evaluation was performed using a Drop CMJ test [23]. This is a one-legged jump from a 20 cm high box down to the floor, followed by an immediate maximum vertical countermovement jump. A light mat, consisting of infrared light beams placed in front of the box, determined the height of the jump. Three to five jumps were performed using each leg and the highest value for both the injured and healthy limb was used.

### Statistical analysis

The sample size was calculated based on the results of the tendon length of the injured limb at 6 and 12 months after injury [25]. It was estimated that a sample of 57 patients was needed to detect a statistically significant difference (*p* < 0.05, power 95%) for the elongation of the Achilles tendon length between 6 and 12 months after injury. Descriptive data are reported as the mean, standard deviation (SD), median and interquartile range (IQR). A comparison between the ATRA, tendon length, HRH and Drop CMJ between the injured and non-injured limb at 6 and 12 months after injury was made using a paired *t* test. The primary outcome for this study is the correlation between tendon elongation, evaluated with US (cm), and the relative ATRA (°) 6 and 12 months after the injury.

The correlations between two continuous variables (relative ATRA, HRH, Drop CMJ, ATRS) were estimated using Spearman’s rho correlation coefficient for non-normally distributed data and Pearson’s correlation for normally distributed data. The distribution was determined by a visual inspection of a histogram and by the finding of a statistically significant *p* value in the Shapiro–Wilk test. As an effect size, *r*^2^ was calculated. The strength of the correlation was determined by the correlation coefficient. A correlation coefficient of > 0.8 was considered very strong, 0.8–0.6 moderately strong, 0.5–0.3 fair and < 3 poor [6]. The level of significance was set at *p* < 0.05. All the data were analysed using IBM SPSS Statistics Version 28.

## Results

Sixty-six subjects were included in this study. 6 patients were excluded; 1 patient declined due to fear of COVID-19, another suffered excessive elongation and was unable to perform the evaluations, 1 patient was incorrectly included (older than the upper age limit) and 3 subjects withdrew from the study after the 6-month evaluation for unknown reasons. The demographics of the study subjects are presented in Table 1.

**Table 1:** Patient demographics presented by the mean (SD) and percentage (*n*)

Variable	Total *n* = 60	Women *n* = 13	Men *n* = 47
Age, years	43 (9)	42 (8)	44
Height, cm	178 (8)	169 (5)	181 (6)
Weight, kg	82 (13)	68 (5)	86 (11)
BMI	25.7 (3.0)	24.0 (2.0)	26.1 (3.1)
Injured side			
Right (*n*)	53% (32)	54% (7)	53% (25)
Left (*n*)	47% (28)	46% (6)	47% (22)
Treatment			
Surgery	35% (21)	38% (5)	34% (16)
Non-surgery	65% (39)	62% (8)	66% (31)

*n* number of patients

There was a significant difference between the injured side compared with the non-injured side at 6 months and 12 months regarding the ATRA, tendon length, HRH and Drop CMJ. The data are presented in Table 2.

**Table 2:** Comparison between the injured and non-injured side at 6 and 12 months presented by the mean (SD)

Variable	6 months			12 months		
	Non-injured side	Injured side	*p* value	Non-injured side	Injured side	*p* value
ATRA^a^ (°)	47.1 (5.7)	52.9 (6.4)	< 0.001	46.6 (6.2)	51.6 (5.6)	< 0.001
Tendon length (cm)	21.4 (2.7)	23.4 (3.1)	< 0.001	21.5 (2.2)	23.1 (2.9)	< 0.001
HRH^b^ (cm)	14.0 (2.4)	10.1 (3.1)	< 0.001	13.9 (2.1)	11.6 (2.1)	< 0.001
Drop CMJ^c^ (cm)	15.6 (4.8)	12.1 (4.6)	< 0.001	15.8 (3.8)	13.1 (3.9)	< 0.001

^a^Achilles tendon resting angle

^b^Heel-rise height

^c^Drop countermovement jump

**Fig. 2:**
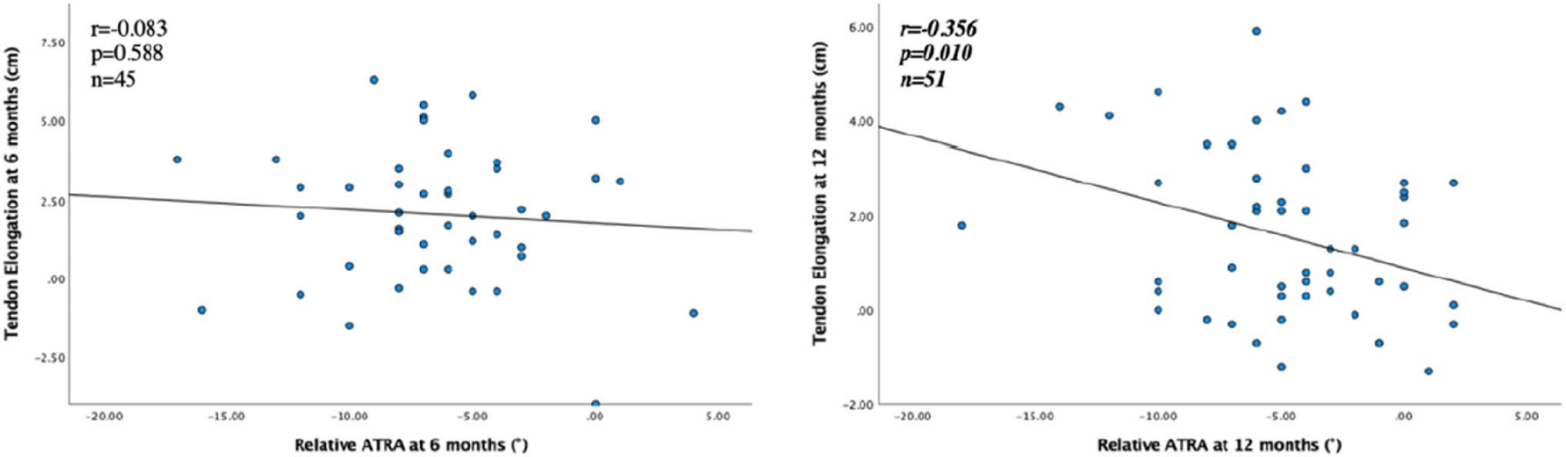
Correlation between tendon elongation (cm) and relative Achilles tendon resting angle (degrees) at 6 months and at 12 months after Achilles tendon rupture. The line represents linear regression model

**Fig. 3:**
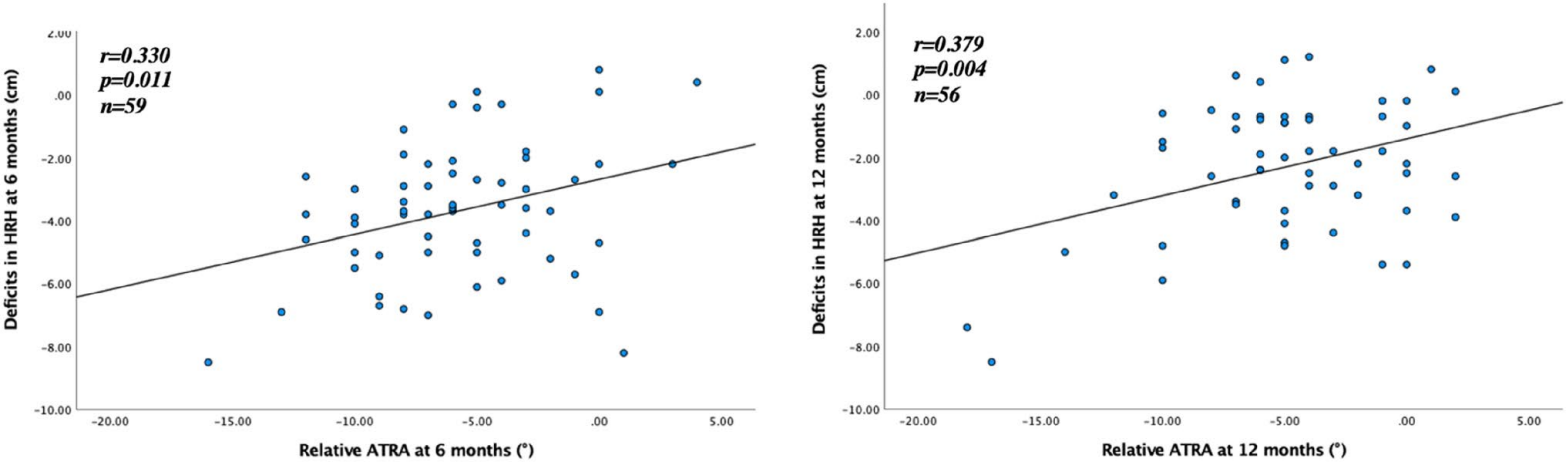
Correlation between deficits in heel-rise height (cm) and relative Achilles tendon resting angle (degrees) at 6 months and at 12 months after Achilles tendon rupture. The line represents the linear regression model

**Fig. 4:**
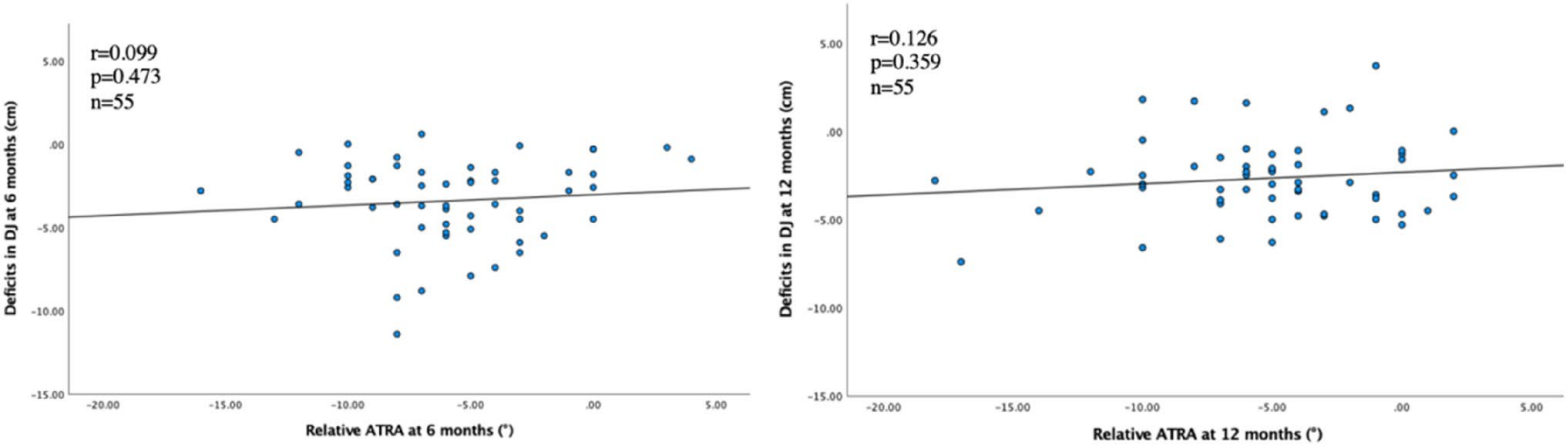
Correlation between deficits in drop countermovement jump (cm) and relative Achilles tendon resting angle (degrees) at 6 months and at 12 months after Achilles tendon rupture. The line represents the linear regression model

**Fig. 5:**
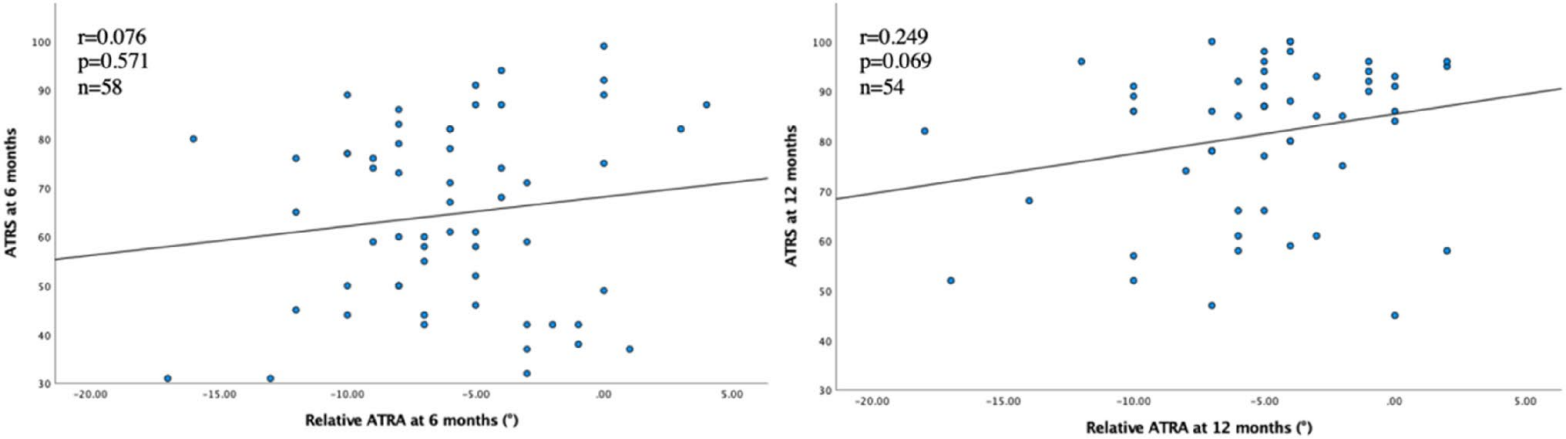
Correlation between Achilles tendon total rupture score and relative Achilles tendon resting angle (degrees) at 6 months and at 12 months after Achilles tendon rupture. The line represents linear regression model

The correlations between the relative ATRA at 6 and 12 months and tendon length, ATRA, HRH, Drop CMJ and ATRS are presented in Figs. 2, 3, 4, 5. A negative correlation (*r* = − 0.356, *p* = 0.010) between the relative ATRA and tendon elongation was seen at 12 months but not at 6 months after ATR (Fig. 2). There was also a positive correlation at both 6 and 12 months between the relative ATRA and HRH (*r* = 0.330, *p* = 0.011; *r* = 0.379, *p* = 0.004) (Fig. 3). There was no correlation between Drop CMJ and the relative ATRA or ATRS and the relative ATRA, at 6 or 12 months after the injury (Figs. 4 and 5).

## Discussion

The most important finding in this study is that, in all outcome measurements (ATRA, HRH, Drop CMJ and tendon length), the injured side had significant deficits compared with the uninjured side at both 6 and 12 months. There was a significant fair negative correlation between the relative ATRA and tendon elongation at 12 months, but not at 6 months after an ATR. Furthermore, there were fair positive correlations between the relative ATRA and deficits in HRH at 6 and 12 months, such as a resting angle in more dorsiflexion related to a greater deficit in HRH.

The results demonstrate that clinical evaluations including HRH, Drop CMJ and tendon length measured by US provide a better full picture of the functional outcome after an ATR, but that the ATRA follows a similar trend, indicating its value to indirect measurements of the presence of tendon elongation. However, the ATRA could not be recommended as a direct surrogate for US for determining Achilles tendon length.

A correlation between the ATRA and tendon length was also reported by Zellers et al. [28], who found a moderate correlation (*r* = 0.491, *p* = 0.001) between the relative ATRA and tendon elongation evaluated with US 12 months after an ATR. However, Zeller et al. [28] used an inclinometer compared with the manual measurement by a goniometer that was used in the present study, so the results may not be completely comparable. Previously, Carmont et al. [3] found a correlation with the absolute ATRA and the HRH limb symmetry index (LSI) at 12 months, but this was not found to be the case at 9 months following injury. These results are in line with ours. It is worth mentioning that we used the deficits in heel-rise height and not the LSI values. Nor did we use the absolute ATRA. In support of our results, Silbernagel et al. [25] found statistically significant differences between the injured and uninjured side after an ATR at 6 and 12 months after injury (mean (SD) at 6/12 months) in both HRH (− 6.1 cm (1.7)/ − 4.1 cm (1.8)) and tendon length measured by US (3.0 (1.2)/2.6 cm (1.4)).

Mortensen et al. [15] and Kangas et al. [12] found that the injured tendon elongates during the healing process during the first months after an ATR, which is in line with Carmont et al.’s [3] finding that the ATRA also increases up to 3 months after the same injury. However, Eliasson et al. [11] reported elongation up to 6 months following a rupture. Tendons have been noted subsequently to shorten a couple of millimetres, a statistically non-significant amount, up to 12 months following injury [12].

There was no significant correlation between Drop CMJ and the ATRA at either 6 or 12 months. This may require further investigation if it is assumed that a greater relative ATRA contributes to poorer plantar flexion power. It has been shown that a vertical jump test may be less applicable in the evaluation of lower limb strength and tendon elongation [14]. Jumping is a complex movement including multiple muscle groups rather than the calf muscles in isolation. Jumping additionally involves other aspects such as balance and co-ordination [14]. It has also been shown that, during jumping, the decrease in work performed at the ankle can be compensated for by increased work performed at the knee and the hip, resulting in no noticeable deficits in jump heights [29].

In this study, there was no significant correlation between the ATRA and patient-reported outcome (ATRS) at 6 or 12 months after ATR. It is important to highlight the fact that it is not uncommonly noticed that patients adapt their everyday life to their limitations [1, 22]. However, Carmont et al. [3] found a positive association with the ATRA and ATRS at 3 and 6 months but not at 9 months following surgery.

All the patients were evaluated by one experienced physiotherapist with a standardised test battery, which is an important strength. One limitation of this study is that the study design did not include a standardised rehabilitation protocol. Another aspect that could be taken into account is the non-random inclusion of patients, which could be a bias, since the patient population might not represent the common population.

The measurement of the ATRA in patients with an ATR is widely used among orthopaedic surgeons and physiotherapists. The great advantages of the ATRA are that only a goniometer is required and the measurement is easy to perform. The findings in this study confirm that, together with other clinical evaluations, the ATRA could be useful in detecting and evaluating tendon elongation in the clinical setting.

## Conclusion

In combination with other clinical measurements, such as HRH and US, the ATRA is a useful clinical tool for indirect measurements of the presence of tendon elongation throughout recovery. However, the ATRA cannot be regarded as a direct surrogate for US.

As a result, tendon elongation after an ATR is not possible to either detect or reject using only one clinical assessment, such as the ATRA.

## Abbreviations

ATR: Achilles tendon rupture
DVT: Deep vein thrombosis
MRI: Magnetic resonance imaging
US: Ultrasonographic
ATRA: Achilles tendon resting angle
PROMs: Patient-reported outcome measures
HRH: Heel-rise height
Drop CMJ: Drop countermovement jump
ATRS: Achilles tendon total rupture score
SU/Mölndal: Sahlgrenska University Hospital/Mölndal
IQR: Interquartile range
